# A fixed-dose randomized controlled trial of olanzapine for psychosis in Parkinson disease

**DOI:** 10.12688/f1000research.2-150.v1

**Published:** 2013-07-09

**Authors:** Michelle J Nichols, Johanna M Hartlein, Meredith GA Eicken, Brad A Racette, Kevin J Black

**Affiliations:** 1 Department of Psychiatry, Washington University School of Medicine, St. Louis MO, 63110, USA; 2 Current affiliation: UT Southwestern Medical Center, Dallas TX, 75390, USA; 3 Department of Neurology, Washington University School of Medicine, St. Louis MO, 63110, USA; 4 Department of Biology, Washington University, St. Louis MO, 63110, USA; 5 Current affiliation: Massachusetts General Hospital, Boston MA, 02114-2622, USA; 6 Department of Radiology, Washington University School of Medicine, St. Louis MO, 63110, USA; 7 Department of Anatomy & Neurobiology, Washington University School of Medicine, St. Louis MO, 63110, USA

## Abstract

**Background:** Psychosis is a common and debilitating side effect of long-term dopaminergic treatment of Parkinson disease (PD). While clozapine is an effective treatment, the need for blood monitoring has limited its first-line use.

**Objective:** Since olanzapine shows similar receptor affinity to clozapine, we hypothesized that it might be an effective alternative to clozapine for treatment of drug-induced psychosis (DIP) in PD, and that lower doses than usual might make it tolerable.

**Methods:** In 1998-2003 we conducted a four-week, double-blind, placebo-controlled, parallel group, fixed-dose trial of olanzapine (0, 2.5mg, or 5mg) in 23 PD patients with DIP while allowing for clinically realistic dose adjustments of dopaminomimetic mid-study. The primary outcome measures were Brief Psychiatric Rating Scale (BPRS) ratings scored from videotaped interviews after study termination by an observer blinded to dose assignment and to interview timing, and CGI (Clinical Global Impression). The Unified Parkinson’s Disease Rating Scale motor subscale (UPDRS) was the primary measure of tolerability.

**Results:** Intention-to-treat analysis found no significant differences among treatment groups in study completion or serious adverse events. However, a disproportionate number of olanzapine vs. placebo subjects reported mild side effects (p<0.04), many citing motor worsening. Fourteen patients completed the study (seven on placebo, two on 2.5mg olanzapine, five on 5mg olanzapine). In study completers, analysis by repeated measures ANOVA revealed no significant difference between olanzapine and placebo groups in BPRS psychosis reduction (p=0.536), parkinsonism (p=0.608), or any other measured parameters (CGI, MMSE, Beck Depression Inventory, Hamilton Depression score, PDQ‑39, Schwab-England ADL assessment, and sleep scores).

**Conclusion:** This study adds to other evidence that olanzapine is ineffective in treating medication-induced psychosis in Parkinson disease.

## Introduction

Drug-induced psychosis (DIP) is a significant and disabling complication of long-term treatment of Parkinson disease (PD), affecting a large minority of PD patients receiving chronic dopaminergic therapy ^1^. Visual hallucinations are the most commonly reported psychotic phenomena in this population, with auditory, tactile, somatic, and olfactory hallucinations being much less common. Delusions, when they occur, often antedate visual hallucinations and commonly are paranoid or persecutory in nature ^2, 3^. In addition to the increased caregiver burden caused by psychosis and its sequelae, hallucinations in the context of chronically treated PD tend to be progressive in nature, resulting in increased propensity for nursing home placement and subsequent higher mortality ^4, 5^. These sobering associations suggest aggressive management of DIP in this population. However, either dose reduction of antiparkinsonian medications or addition of traditional neuroleptics usually increases parkinsonian motor disabilities. Atypical antipsychotics, with their comparatively lower incidence of parkinsonism in schizophrenia, have potential advantages for treatment of hallucinations in this sensitive population ^1^.

Until recently, the only treatment proven with randomized, placebo-controlled studies to reduce DIP has been clozapine, an agent that does not worsen motor function ^6– 8^. Despite these favorable data, use of clozapine has been limited secondary to its rare but potentially serious risk of agranulocytosis and the consequent necessity for frequent blood draws ^1^. Thus alternative treatments have been eagerly sought.

Quetiapine has become the most commonly prescribed antipsychotic in DIP ^9^. Although double-blind, placebo-controlled trials of quetiapine in PD confirmed it is well tolerated in terms of motor side effects, it has not proven significantly more effective than placebo in treating psychosis ^10– 15^, and a head-to-head comparison found clozapine superior to quetiapine ^16^. Ziprasidone showed some benefit in open-label experience ^17^, including in a random-assignment open comparison to clozapine ^18^. However, ziprasidone can cause motor side effects in PD and is not generally considered standard therapy for DIP ^1, 19^. Other treatments, such as ondansetron, acetylcholinesterase inhibitors, and electroconvulsive therapy are supported by limited data in idiopathic Parkinson disease but are generally not viewed as first-line therapy ^1, 19^. Recently, a phase III clinical trial of a serotonin 5HT _2A_ inverse agonist, pimavanserin, showed benefit over placebo, but the drug will not be available in the U.S. at least until late 2014 ^20– 22^.

Clozapine’s antipsychotic efficacy is often attributed to its D _4_ receptor antagonism. It is also posited that its robust 5HT _2A_ receptor antagonism, especially in relation to its relatively weaker D _2_ receptor blockade, actually increases dopamine transmission in prefrontal cortical and nigrostriatal projections ^23^. This may account for the cognitive improvement as well as paucity of extrapyramidal adverse events observed in clozapine-treated patients with dopaminomimetic-induced psychosis ^23, 24^. Olanzapine, therefore, with its ostensibly similar receptor binding profile to clozapine at D _2_, D _4_, and serotonergic receptors (especially 5HT _2A_ and 5HT _2C_), and muscarinic sites, provides a theoretically encouraging alternative to clozapine in this fragile population ^25^.

An initial open study of olanzapine in Parkinson disease revealed antipsychotic benefit without motor deterioration when drug dosage was optimized in a slow titration (mean daily dose at end of study was 6.5mg) and dopaminomimetic dose adjustments were allowed ^26^. Aarsland and colleagues replicated these findings in a relatively more challenging population of Parkinson disease patients with and without dementia ^27^. Several other small, open-label studies of olanzapine, however, have demonstrated antipsychotic benefit but at the expense of intolerable worsening of gait and bradykinesia, frequently leading to premature termination of the drug ^28– 30^. Another small open-label trial and case report series suggested unacceptable Parkinsonian motor deterioration in the context of dubious antipsychotic efficacy ^31, 32^. Later, two double-blind placebo-controlled trials revealed equivocal antipsychotic benefit and problematic motor decline in PD patients with DIP treated with 2.5–15mg/day olanzapine (mean final doses 4.1–4.6mg/day) ^33, 34^. As a result, experts have recommended against the use of olanzapine in PD ^1, 19^.

None of these studies, however, were parallel-group fixed-dose trials, and some allowed for neuroleptic dose in the same range as approved for schizophrenia; experience with clozapine suggests that an effective antipsychotic dose in PD is often an order of magnitude less than that typical for schizophrenia treatment. In addition, the two double-blind placebo-controlled trials did not permit adjustments of subjects’ dopaminomimetics, which might have alleviated motoric side effects. Finally, some of the studies cited were terminated prematurely due to side effects. Given that the only marketed drug for which efficacy has been shown is clozapine, demonstrating efficacy for an alternative agent would be important, and a fixed low dose of olanzapine (2.5mg/day) may allow a reasonably low incidence of side effects if dopaminomimetic dose adjustments are allowed. We discuss here the findings of a double-blind, placebo-controlled study of fixed, low-dose olanzapine for treatment of DIP in the context of flexible dopaminomimetic dosing. The hypothesis was that olanzapine given in this fashion would reduce DIP in patients with idiopathic PD significantly more than would a placebo, without causing intolerable motor worsening.

## Methods and materials

The completed CONSORT checklist ^35, 36^ and the original study protocol are available in the Data Files.

### Ethics statement

All patients gave written informed consent to participate in the study, which was approved by the Washington University Human Studies Committee (approval # 97-0366). In most cases an appropriate surrogate decision maker also consented. FDA approval was through IND # 53,556. This trial concluded in 2003, so it is exempt from the current ICMJE requirement of prospectively registering clinical trials.

### Patient selection

Twenty-four patients were recruited from the Washington University Movement Disorders Center from February 1998 to October 2003. Patients were examined by a movement disorders specialist and diagnosed with idiopathic PD based on presence of at least two of three cardinal manifestations of the disease (rigidity, bradykinesia, rest tremor), response to levodopa or a dopamine agonist, and absence of historical or examination features suggesting secondary parkinsonism. Subjects were treated with levodopa and were experiencing clinically significant hallucinations or delusions, as judged by their treating neurologist or psychiatrist and by the investigator (KJB). Subjects were required to be over 30 years old and have a caregiver who could provide a reliable report. At study entry, patients were required to be treated with the lowest clinically acceptable dose of dopaminomimetic. Patients treated only with a dopamine agonist were not entered in the study, as it was deemed more clinically appropriate to try a switch to levodopa before adding an antipsychotic. Exclusion criteria included a Folstein Mini-Mental State Examination (MMSE) score < 22 ^37^, pregnancy, concurrent diagnosis of delirium (unless clearly explained by dopaminomimetics), catatonia or neuroleptic malignant syndrome (NMS)-like syndrome, other confounding central nervous system (CNS) illness or systemic illness with potential CNS effects, antipsychotic use within the last month predating study enrollment (within the past six months for depot neuroleptics), history of olanzapine sensitivity, or any expectation of significant medical or surgical intervention within six weeks after enrollment. Subjects were also excluded if severity of psychosis warranted hospitalization or if, in the investigator’s judgment, psychosis severity would have made randomization to placebo inappropriate.

### Treatment protocol

Patients were randomized 1:1:1 to treatment with placebo or either of two doses of olanzapine. At study initiation, treatment groups consisted of a placebo arm, a 5mg arm, in which patients received this dosage nightly throughout the four weeks of investigation, and a 10mg arm, in which patients received 5mg for the first week and 10mg thereafter. Subjects received matched tablets or capsules provided by Lilly Research Laboratories (Indianapolis, IN), who provided the investigator with sealed, sequentially numbered envelopes containing the medication identity for each subject. The envelopes were not opened until after all data were collected and reviewed for accuracy, and after all decisions about statistical analysis were final, so that both investigators and patients were blind to intervention assignment. The randomization was done by Lilly. KJB enrolled subjects and patients were assigned to treatment packages sequentially by enrollment date.

After the first five patients were enrolled, an interim safety analysis was conducted by a reviewer otherwise not involved in the study, in light of reports published since the study initiation that higher olanzapine doses caused intolerable exacerbation of parkinsonism in PD. Though serious adverse events were no more common in the treatment groups than in the placebo group, it was decided at this time that the two active treatment arms would be changed to fixed doses of 2.5mg and 5mg olanzapine, maintained throughout the four weeks of study. New treatment packages were received and the blind was maintained until after data analysis, as above. No other changes to the protocol were made. See Table 1 for a summary of the final study design. The study was planned for 10 subjects in each of three dose arms. This would produce 90% power (at alpha = 0.05) to detect a change of the magnitude and variability seen in the Wolters *et al.* ^26^ report.

**Table 1.** Summary of final study design.

Baseline	Weeks 1–2	2 week visit	Weeks 3–4	4 week visit
Clinical evaluation; randomize	Placebo 2.5mg 5mg	Clinical evaluation; ↑ dopaminometic, if indicated	Placebo 2.5mg 5mg	Clinical evaluation; return to routine clinical care

**Figure 1.**
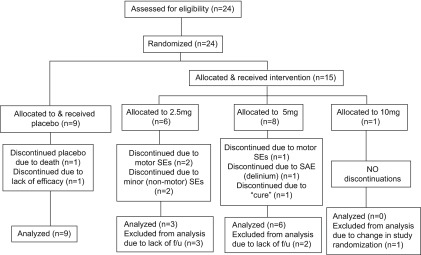
CONSORT flowchart.

This table summarizes the study design and timing of assessments and interventions for the last 19 subjects enrolled in the study. ↑ dopaminometic: dose increase allowed for antiparkinsonian medication, if parkinsonism had worsened since starting the study. See Methods and Figure 1 for further details.

Subjects received a baseline evaluation that involved a full psychiatric, neurologic, and medical history and examination, CGI (Clinical Global Impression) by MD ^38^, PDQ-39, a self-rated quality-of-life measure for PD ^39^, videotaped interview for later BPRS (Brief Psychiatric Rating Scale) rating blind to drug dose and blind to which visit was being rated ^40^, Schwab-England ADL assessment ^41^, UPDRS (Unified Parkinson’s Disease Rating Scale), section III (motor) ^42^, MMSE ^37^, HDRS (Hamilton Depression Rating Scale) ^43^, BDI (Beck Depression Inventory) ^44^, and patient/caretaker reported hours and quality of sleep. Repeated measures at the two-week interim visit and the final four-week evaluation included CGI (by MD, patient, and caretaker), videotaped interview for later blinded BPRS, Schwab-England ADL assessment, UPDRS, MMSE, PDQ-39, BDI, sleep questionnaire, and pill counts. All assessments were done at Washington University Medical Center.

Primary efficacy measures were CGI scores and BPRS ratings of psychosis. At each visit, the coordinator interviewed the patient during videotaping using a semi-structured interview designed to facilitate later scoring of psychopathology using the BPRS ^40, 45, 46^. After all subjects had completed participation, the videotaped segments were edited to remove references to date or study visit. Author KJB in consultation with a BPRS expert (John G Csernansky, MD) wrote rules for rating “motor retardation” and other BPRS items potentially influenced by parkinsonism (see Supplementary materials), and trained author MJN in BPRS ratings. Videotaped segments were reviewed in random order by MJN, who was unaware of drug assignment or treatment duration at the time of the visit. BPRS ratings used the anchored BPRS and each item was scored from 1–7 ^40^. Secondary efficacy measures included the PDQ-39, ADL assessments (Schwab-England and UPDRS), BDI, and sleep log. Primary safety measures were UPDRS motor ratings, sleep logs, and MMSE in addition to clinical review of systems.

### Statistical analysis

Prior to unblinding of drug codes, the decision was made to analyze data from weeks 0–2 and weeks 2–4 separately. This *a priori* decision was made since adjustment of dopaminomimetics was allowed at the interim (week 2) visit. Change from 0 to 2 weeks was chosen to be the primary test of efficacy. An intention-to-treat (ITT) analysis was performed on all enrolled subjects. However, since some subjects dropped out without completing outcome measures at a follow-up visit, the ITT analysis was limited to between-group comparisons of dropout rate, serious adverse events, and reported worsening of parkinsonism or other side effects judged to be at least mild in severity. Adverse events, side effects, and study withdrawal were compared between groups using the chi-squared test.

For those subjects with data at both time points of an epoch, primary and secondary efficacy measures were tested separately for the two epochs using repeated-measures ANOVA to compare the groups. The decision was made *a priori* to include any subject in these analyses if that patient had taken at least one week’s worth of drug during an epoch and returned for a follow-up visit. A secondary *post hoc* analysis of the data from trial completers was also performed across all three visits using repeated-measures ANOVA. Statistical computations used STATISTICA 7.1 (StatSoft, Inc., Tulsa, OK) or Excel (Microsoft, Redmond, WA).

## Results

### Baseline characteristics

A total of 24 patients were enrolled (see Figure 1). Though the original study design sought enrollment of 30 patients, the study was terminated early, secondary to the growing body of literature questioning the safety of olanzapine in the treatment of DIP as well as the increasing difficulty in enrolling antipsychotic-naive patients.

Only one subject was treated with 10mg (one other was randomized to the 10mg group, but was treated only for one week, so received only 5mg doses). His hallucinations were rated “very much improved” at the study end; he required no adjustment in dopaminomimetic dose mid-study and no side effects were observed. This 10mg subject was not included in statistical analyses. In the remaining 23 subjects, no significant imbalances were present at baseline between placebo and treatment groups on any demographic characteristic or any psychiatric or neurologic measure ( Table 2).

**Table 2.** Patient characteristics at baseline.

		Olanzapine		
Measure	Placebo (n=9)	2.5mg (n=6)	5mg (n=8)	p value
Age	71.3 (6.5)	70.7 (8.1)	72.4 (4.8)	0.882
MMSE	26 (2.6)	27 (3.6)	27 (2.7)	0.976
BPRS-T	34.8 (5.9)	34.3 (5.4)	33.4 (3)	0.874
BPRS-P	7.9 (2)	9 (3)	7.8 (2.1)	0.633
UPDRS, motor score	30 (11)	27.5 (13.1)	31 (11.6)	0.855
PDQ-39	53 (25.7)	59 (15.9)	59 (27.3)	0.867
BDI	10.1 (6)	9.8 (6)	12.6 (9.2)	0.738
HAM-D	8.7 (6.1)	5.3 (1.6)	11.6 (7.6)	0.177
CGI	4.1 (0.9)	3.2 (1)	3.9 (0.8)	0.161
INS	4.2 (4)	4 (2.1)	2.6 (2.6)	0.566
HYPINS	1.5 (1)	2.3 (1.9)	2.6 (2.1)	0.446
SEADL	76 (15)	72 (24)	75 (17)	0.918

Values are given as mean (SD). MMSE, Folstein mini mental test examination; BPRS-T, Brief Psychiatric Rating Scale total score; BPRS-P, psychosis subscale; UPDRS, Unified Parkinson’s Disease Rating Scale; PDQ-39, Parkinson’s disease quality of life questionnaire; BDI, Beck depression inventory; HAM-D, Hamilton depression rating scale; CGI, Clinical global impression; INS, Insomnia score; HYP, Hypersomnia score; SEADL, Schwab-England ADL assessment.

### Intention-to-treat analyses

The intention-to-treat analyses did not show significant differences between groups except for incidence of mild side effects (p<0.04) ( Table 3). While spontaneous report of motor side effects was not statistically significant between groups, a disproportionate number of olanzapine vs. placebo group subjects who withdrew did so secondary to reported motor side effects (0% of placebo withdrawers vs. 21% of olanzapine withdrawers). Nine subjects did not complete the study: two from the placebo group, four from the 2.5mg olanzapine group, and three from the 5mg olanzapine group. In the placebo group, one patient died of myocardial infarction and another withdrew from the study secondary to lack of efficacy. In the 5mg olanzapine group, two reported serious adverse events and a third discontinued her medication following the first dose, declaring herself “cured”. Of the 5mg subjects who withdrew for serious adverse events, one was hospitalized with delirium three weeks into the study; the other withdrew after day six due to hospitalization with hip fracture and pneumonia, and reported worsening PD symptoms prior to dropout. Of the four subjects who dropped out of the 2.5mg olanzapine group, two withdrew due to worsening parkinsonian symptoms, one secondary to unspecified side effects, and one secondary to “feeling confused”. Only two subjects in the 2.5mg group completed the study, both requiring increases of their levodopa dose at their interim visit. One each in the placebo and 5mg olanzapine arms also required levodopa adjustment at their two-week assessment. Retention and attrition of study subjects is summarized in Table 3 and Figure 1.

**Table 3.** Subject retention and side effects by group.

		Olanzapine			
	Placebo	2.5mg	5mg	All	p value
# enrolled	9	6	8	23	
# withdrew	2 (22%)	4 (66%)	3 (38%)	9 (39%)	0.2232
# withdrew for motor SEs	0 (0%)	2 (33%)	1 (12%)	3 (13%)	0.1712
# w/motor SE complaint	1 (11%)	2 (33%)	1 (12%)	4 (17%)	0.4863
# w/any mild SEs	2 (22%)	5 (83%)	2 (25%)	9 (39%)	*0.0356
# w/serious adverse events	1 (11%)	0 (0%)	2 (25%)	3 (13%)	0.3795
# included in 1st epoch	9 (100%)	3 (50%)	5 (63%)	17 (74%)	0.0640
# included in 2nd epoch	7 (78%)	2 (33%)	5 (63%)	14 (61%)	0.2232
# w/dopaminomimetic ↑	1 (11%)	2 (33%)	1 (13%)	4 (17%)	0.4863

Side effects (SEs) were any complaint of drug spontaneously reported by the patient, independent of whether SE intensity was severe enough to prompt withdrawal from the study. Serious adverse events always prompted withdrawal. SE, side effects; ↑, increase; 1st epoch, week 0–2 analysis; 2nd epoch, week 2–4 analysis, *, p<0.05.

To assess adequacy of blinding, both the primary investigator and study subjects were asked on study completion (or drop-out) to guess the identity of administered medication (i.e., olanzapine vs. placebo). Both investigator and patient were much more likely than chance would predict to correctly guess the identity of administered medication (for investigator, χ ^2^=12.29, p=0.0021; for study subjects, χ ^2^=6.94, p=0.0312). However, the videotape rater had no information about side effects.

Negative results from a randomized controlled trial of olanzapine for psychosis in Parkinson disease: data, CONSORT checklist and initial study protocol CONSORT checklist
Subject characteristics at study entry: S#, subject ID for this study; DRUG, olanzapine dose (mg) this patient took; ADJUST, whether the dose of antiparkinsonian medication was adjusted at the week 2 visit. For remaining columns, the trailing zero in the column heading refers to the score at the baseline (week 0) visit; see Methods section for references. BPRST, BPRS total score; BPRSP, BPRS psychosis items subscore; PDQ, PDQ39 score; BDI, Beck Depression Inventory; HamD, Hamilton Depression Rating Scale; CGIMDo/a, Clinical Global Impression score for overall clinical state as rated by investigator; INS, insomnia score from sleep rating scale; HYPIN, hypersomnia score from sleep rating scale; SEADLS, Schwab-England score; BlindKB, investigator guess at week 4 as to drug assignment; BlindPt., patient guess at week 4 as to drug assignment.
Outcomes other than blinded BPRS ratings: S#, subject ID for this study; DRUG, olanzapine dose (mg) this patient took; ADJUST, whether the dose of antiparkinsonian medication was adjusted at the week 2 visit; change, details of that change; use0-2, analyze this subject’s data in the ANOVA for weeks 0-2; use2-4, analyze this subject’s data in the ANOVA for weeks 2-4; WD, ended study participation early; WDSE, withdrew from study because of side effects; WDnowork, withdrew from study because of lack of benefit; WDcure, withdrew from study because subject pronounced self “cured” after one dose; SAEs, serious adverse events; mild SEs, mild side effects; other, other comments on efficacy or side effects; handed, right- or left-handed. For remaining columns, the trailing numeral in the column heading refers to the score at the visit from week 0, 2, or 4. See Methods section for additional information. VH, visual hallucinations present; AH, auditory hallucinations present; Del, delusions present; BPRST, BPRS total score; BPRSP, BPRS psychosis items subscore; 1UPDRS, UPDRS subscale 1; 2UPDRS, UPDRS subscale 2 (etc.); PDQ, PDQ39 score; BDI, Beck Depression Inventory; HamD, Hamilton Depression Rating Scale; CGI, Clinical Global Impression scale; CGIMD, CGI rated by investigator; CGI…overall, CGI score for overall clinical state; CGIPT, CGI rated by patient; CGI…hall, CGI for hallucinations; CGI…improve, CGI improvement from study initiation; INS, insomnia score from sleep rating scale; BlindJH, study RN guess at week 4 as to drug assignment; BlindKB, investigator guess at week 4 as to drug assignment; BlindKBdrug, same, collapsed to simply olanzapine vs placebo (no dose category); BlindPt., patient guess at week 4 as to drug assignment; BlindBR, guess of study neurologist at week 4 as to drug assignment; 0/5/10, whether this subject was enrolled under the initial study drug assignment (placebo vs 5 or 10mg); HYPIN, hypersomnia score from sleep rating scale; SEADLS, Schwab-England score.
Blinded BPRS ratings from videotape: video ID#, code by which blinded videotape reviewer scored each video segment; BPRS-T, BPRS total score; BPRS-P, BPRS psychosis items subscore.

### Primary planned analyses

**Figure 2.**
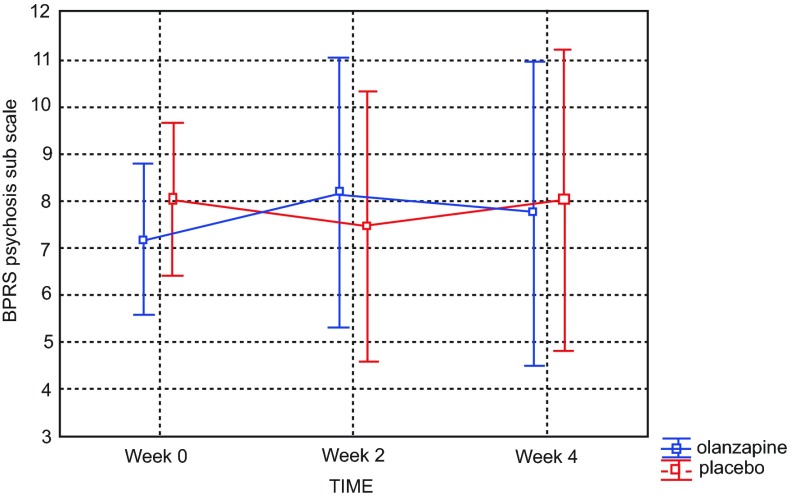
Brief Psychiatric Rating Scale (BPRS) scores across four week study revealed no significant difference between placebo and olanzapine groups among study completers. Current effect: F(2, 24)=0.64064, p=0.53573. Effective hypothesis decomposition. Vertical bars denote 0.95 confidence intervals. Olanzapine-blue; placebo-red.

Analysis of the psychosis subscale of BPRS scores (the more sensitive of our primary efficacy measures) did not reveal a statistically significant difference between groups (drug doses) in severity of psychosis in either the week 0–2 epoch (p=0.433) or the week 2–4 epoch (p=0.393). Again, *post hoc* analysis in study completers revealed no statistical significance in psychosis reduction between olanzapine (combined groups) and placebo (p=0.536), as shown in Figure 2.

**Figure 3.**
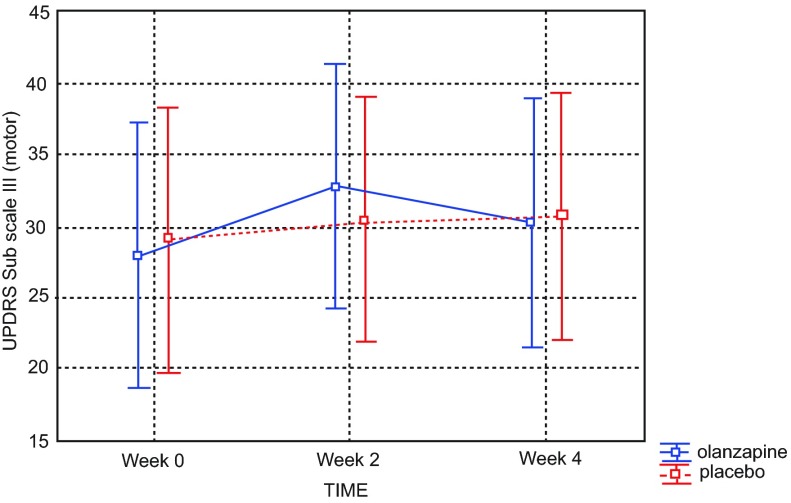
Unified Parkinson’s Disease Rating Scale (UPDRS) scores across four week study revealed no significant difference between placebo and olanzapine groups among study completers. Current effect: F(2, 24)=0.50826, p=0.60787. Effective hypothesis decomposition. Vertical bars denote 0.95 confidence intervals. Olanzapine-blue; placebo-red.

Data from the first and second epochs revealed no statistically significant difference in parkinsonian signs across treatment groups, as measured by the UPDRS III (week 0–2 epoch, placebo vs. 2.5mg olanzapine group p=0.172; week 2–4 epoch p=0.677). *Post hoc* analysis of UPDRS motor scores comparing olanzapine (combined groups) versus placebo across the duration of study found no significant difference in parkinsonism among study completers (p=0.608) ( Figure 3).

Analyses were repeated in like fashion for all other psychiatric and neurological parameters (CGI impression, CGI improvement, BPRS total, BDI, MMSE, insomnia score, hypersomnolence score, PDQ-39, and Schwab-England ADL assessment), none of which revealed statistical significance between olanzapine groups and placebo.

## Discussion

The study failed to reject the null hypothesis. This could be a Type II error, but larger studies of olanzapine also failed to demonstrate antipsychotic efficacy of this drug in the PD population ^14, 33^. In study completers, we did not observe the motoric exacerbation documented in several studies in the literature ^28– 34^, but perhaps this is a function of our allowance for dopaminomimetic increase mid-study as well as a selection bias in some analyses for those subjects who best tolerated the medication and therefore completed the study. After all, of the nine subjects who withdrew from the study, a third identified a worsening of their motor disability prior to dropout, all of whom were discovered on unblinding to have been randomized to olanzapine. Therefore the good retrospective accuracy of investigator and patient guesses of study drug identity is not surprising.

The subjects enrolled are relatively typical of PD patients with psychotic symptoms with a few exceptions. Subjects with urgent need for treatment were not enrolled for ethical reasons. Although mild dementia was allowed, this sample had relatively high cognitive functioning, with a mean MMSE score > 26 ( Table 2). Finally, at this center, some of the patients are referred for subspecialty movement disorders consultation, though a large fraction of the patients are not referred and are typical of PD patients treated in the community. With these caveats, the results appear to be generally applicable to patients with PD and psychosis.

One methodological innovation in this study was the use of videotape to record semi-standardized interviews for later analysis by a rater blind not only to drug assignment but also to time (i.e., week 0, week 2, or week 4). The rationale was to minimize rater expectation of improvement over time that might reduce our power to detect significantly greater improvement in the active treatment groups. It also reduced the likelihood of rater unblinding.

This trial supports other evidence suggesting that olanzapine is ineffective for relieving dopaminomimetic-induced psychotic symptoms in Parkinson disease and that it may cause intolerable worsening of motor disability ^1, 19^. This trial also underscores the importance of rigorous study design for the assessment of drug effectiveness in special populations, as we and others have not replicated the early, positive open-label experience reported for olanzapine in this population. If clozapine’s prominence in the clinical management of DIP in PD is to be usurped, antipsychotic agents will have to meet the burden of proof of double-blind, randomized, placebo-controlled trials.

## Supplementary materials

**Guidelines for rating selected BPRS items in a treatment study of psychosis in Parkinson disease.**

1. Emotional withdrawal = interpersonal relatedness during interview.

2. Tension:

    a. Ignore: rest tremors, postural tremors, chorea, athetosis, dystonia.

    b. Include: tardive dyskinesia and akathisia.

3. Depressive mood rating does *not* consider “pure apathy” ( *i.e.*, apathy w/o other depressive signs or symptoms), but apathy *can* contribute to the total judgment of depressive mood if other signs or symptoms are present.

4. Hallucinatory behavior:

    a. 2 = illusions and “shadow in the corner of the eye”.

    b. 3 = *e.g*., colors on the wall.

    c. ≥ 4 = definitively abnormal sensory perceptions.

5. Motor retardation: Speed of movement, not amplitude (also, depressive retardation is not substantially helped by external cues; if slowed movement is substantially helped by external cues, then it may be more parsimoniously attributed to PD).

6. Unusual thought content: Ratings ≥ 5 require action on delusion.

7. Blunted affect: Rate according to scale, considering emotional variance, regardless of amplitude; remember that flat/blunted affect is not equivalent to depressed affect.

8. Disorientation: Off by one day of week = 3.

Motor hyperactivity: Limit rating to pressured speech and voluntary movement; festination does not count.

Kevin J Black MD consulted with John G Csernansky MD to write these additional rules for scoring BPRS items potentially influenced by motor signs in Parkinson disease patients.
