# Supplementary material for: A fixed-dose randomized controlled trial of olanzapine for psychosis in Parkinson disease
Source: F1000Res. 2013 Jul 9;2:150. [Version 1] doi: 10.12688/f1000research.2-150.v1 (PMC3907164; doi:10.12688/f1000research.2-150.v1)
Supplement: Negative results from a randomized controlled trial of olanzapine for psychosis in Parkinson disease: data, CONSORT checklist and initial study protocol — CONSORT checklist Subject characteristics at study entry: S#, subject ID for this study; DRUG, olanzapine dose (mg) this patient took; ADJUST, whether the dose of antiparkinsonian medication was adjusted at the week 2 visit. For remaining columns, the trailing zero in the column heading refers to the score at the baseline (week 0) visit; see Methods section for references. BPRST, BPRS total score; BPRSP, BPRS psychosis items subscore; PDQ, PDQ39 score; BDI, Beck Depression Inventory; HamD, Hamilton Depression Rating Scale; CGIMDo/a, Clinical Global Impression score for overall clinical state as rated by investigator; INS, insomnia score from sleep rating scale; HYPIN, hypersomnia score from sleep rating scale; SEADLS, Schwab-England score; BlindKB, investigator guess at week 4 as to drug assignment; BlindPt., patient guess at week 4 as to drug assignment. Outcomes other than blinded BPRS ratings: S#, subject ID for this study; DRUG, olanzapine dose (mg) this patient took; ADJUST, whether the dose of antiparkinsonian medication was adjusted at the week 2 visit; change, details of that change; use0-2, analyze this subject’s data in the ANOVA for weeks 0-2; use2-4, analyze this subject’s data in the ANOVA for weeks 2-4; WD, ended study participation early; WDSE, withdrew from study because of side effects; WDnowork, withdrew from study because of lack of benefit; WDcure, withdrew from study because subject pronounced self “cured” after one dose; SAEs, serious adverse events; mild SEs, mild side effects; other, other comments on efficacy or side effects; handed, right- or left-handed. For remaining columns, the trailing numeral in the column heading refers to the score at the visit from week 0, 2, or 4. See Methods section for additional information. VH, visual hallucinations present; AH, auditory hallucinations present; Del, delusions present; BPRST, BPRS total score; BPRSP, BPRS psychosis items subscore; 1UPDRS, UPDRS subscale 1; 2UPDRS, UPDRS subscale 2 (etc.); PDQ, [file f1000research-2-1686-s0000.tgz › Protocol.pdf]

Double-blind, placebo-controlled, fixed-dose, parallel group study of olanzapine for treatment of psychosis in patients with Parkinson's disease

Kevin J. Black, M.D., and Joel S. Perlmutter
revised May 21, 1997

I. Introduction / Rationale

Psychosis is a common complication of dopamimetic treatment in Parkinson's disease, often limiting treatment of the parkinsonian motor symptoms. However, in this patient population treatment with antipsychotics, including newer agents such as risperidone and remoxipride, is generally disappointing because of intolerable motor side effects. Clozapine is thought to be an effective and generally well-tolerated treatment but does carry the important and unpredictable risk of agranulocytosis in 1%-2% of patients, some of whom may die as a result. Furthermore, there are no adequate prospective studies of clozapine in this population. These points are briefly reviewed elsewhere (Wolters et al., 1996).

Olanzapine is a recently marketed antipsychotic agent which, in studies of patients with psychosis (mostly schizophrenia), caused acute extrapyramidal side effects much less frequently than did the comparison drug haloperidol, but had similar or better efficacy. This suggests it may be better tolerated than other antipsychotic agents in patients with Parkinson's disease and psychosis.

There is one published open study of olanzapine in this population. Olanzapine was generally well-tolerated over the duration of the study, improved psychotic symptoms significantly as measured by the BPRS, and allowed a further increase in dopamimetics (and a consequent improvement in motor functioning) without marked exacerbation of psychosis (Wolters et al., 1996). However, the design of this preliminary study markedly limits the generalizability of its conclusions. Specifically, there was no placebo arm, and raters were aware both of treatment condition and duration of treatment, which may introduce substantial rater bias. Thus the efficacy of olanzapine is not definitively established in this special population.

The present study is intended to test the conclusions of the Wolters et al. study (1996) using a more rigorous approach, and extend the study questions.

II. Objectives

<u>Primary</u>

1) Determine whether olanzapine has superior efficacy to placebo in treating psychosis in patients with Parkinson's disease (PD) who are being treated with dopamimetics.
2) Determine whether in this setting a daily (qhs) dose of 10mg is more effective than a daily dose of 5mg.

<u>Secondary</u>

3) Compare tolerability of 5mg and 10mg daily doses of olanzapine in this population.
4) Determine whether olanzapine treatment of psychosis in Parkinson's disease allows patients to take higher doses of levodopa without exacerbating psychosis.

III. Investigational Plan

A. Summary of Study Design

Patients will be screened and if they meet the criteria given in section III.D.1. will enter into one of three arms, shown as three rows of the table below. Each arm will consist of nightly

(qhs) dosing of matched tablets or capsules containing 0, 5, or 10 mg of olanzapine. However, patients in the 10 mg arm will take only 5 mg nightly for the first week.

See the table below for a diagrammatic summary of the study design.

baseline	1st week	2nd week	2-wk visit	weeks 3-4	4-wk visit
clinical evaluation; randomize	placebo	placebo	clinical evaluation; increase dopamimetic if indicated	placebo	clinical evaluation; return to routine clinical care
	5mg	5mg		5mg	
	5mg	10mg		10mg	

<u>Baseline evaluation:</u>

Psychiatric, neurologic and general medical history and examination; Clinical Global Impression (CGI) by MD, PDQ39 (a brief, self-rated overall quality of life questionnaire), videotaped psychiatric interview for later blinded BPRS (Brief Psychiatric Rating Scale) rating, Schwab-England ADL assessment, full UPDRS (Unified Parkinson's Disease Rating Scale), MMSE (Folstein Mini-Mental State Examination), HDRS (Hamilton Depression Rating Scale), Beck depression inventory (Beck), patient- or caretaker-reported sleep hours/quality

<u>Interim evaluation:</u>

clinical evaluation; CGI (MD, pt, caretaker), videotaped psychiatric interview for later blinded BPRS, Schwab-England, full UPDRS, MMSE, pill counts, PDQ39, Beck, sleep hours

<u>Final evaluation:</u>

clinical evaluation; CGI (MD, pt, caretaker), videotaped psychiatric interview for later blinded BPRS, Schwab-England, full UPDRS, MMSE, pill counts, PDQ39, Beck, sleep hours

B. Rationale for Design and Control

Our design can significantly strengthen the conclusions of Wolters et al. (1996) in three ways. First, with the parallel group, placebo-controlled design, we can show whether olanzapine is superior to placebo in treating PD psychosis. Second, with a fixed dose design we can address the question of optimal olanzapine dose in these patients. Third, the Wolters et al study did not use blinded ratings of psychosis. To address this, we will videotape clinical interviews at each study visit. The main outcome measure, BPRS ratings of psychosis, can then be rated after the end of the study by an investigator who will be blind not only to olanzapine dose but also to duration of treatment (0, 2, or 4 weeks). This will allow us to show that improvement in any treatment group is not due to investigator or patient expectation of improvement, and may also increase our power as the placebo group may show less change.

We chose 4 weeks rather than a longer study period because clinical experience and the Wolters et al. trial both suggest that improvement in psychosis is usually seen by this point.

The doses chosen are due in part to the desire to bracket the mean final dose in the open study (7mg/day), and in part to the fact that olanzapine is marketed in 5mg and 10mg tablets.

A placebo control is not only scientifically needed but also ethically justified, given the potential side effects of either olanzapine or other treatments of psychosis in this population (beyond the conservative measures we require for study entry).

C. Investigator Information

Please see the CVs submitted separately.

1. Responsibility for Authorship of Clinical Study Report

The principal investigator, Kevin J. Black, M.D., will author the study report.

D. Study Population

1. Criteria for Enrollment

a. Inclusion Criteria

- age 30 years and above
- male or female
- clinical diagnosis of idiopathic Parkinson's disease
- currently experiencing hallucinations, delusions or formal thought disorder (*i.e.* score > 3 on one of the corresponding BPRS items)
- current treatment with dopamimetics
- dopamimetic dosing already at lowest clinically acceptable level (due to needed mobility, etc.)
- availability of family member / caretaker who can reliably report symptoms
- ability to give informed consent, or appropriate substituted consent available
- native or near-native command of English

b. Exclusion Criteria

- MMSE score < 20
- current clinical diagnosis of delirium, unless clearly related only to dopamimetics
- NMS-like syndrome or catatonia
- other concurrent CNS illness, *e.g.* stroke
- current systemic illness likely to affect the CNS, *e.g.* lupus or untreated hypothyroidism
- significant surgical or general medical intervention expected within the next 6 weeks
- history of sensitivity to olanzapine
- antipsychotic use within the last month (6 months for depot neuroleptics)
- pregnancy

2. Sample Size and Statistical Justification

Sample size will be 10 patients per arm (30 total). Using the Wolters et al baseline data as a rough estimate of variance, a power analysis shows that we would have a 90% likelihood of detecting a 50% improvement in total BPRS scores (at alpha = 0.05) within a single treatment group of 10-11 patients. From the same data, power would be slightly higher using the BPRS "positive item" subscale (i.e., the 4 items which reflect hallucinations, delusions, or formal thought disorder).

To rigorously estimate power for our study design would require an estimate of the variance in the *change* of BPRS scores with olanzapine (or placebo). We do not have that data. However, we suspect on clinical grounds that there will be no meaningful improvement in the placebo group (here is where the blinded videotape ratings may add to our power), so we expect to also find a significant difference between groups, using a comparable number of patients per treatment arm. Efficacy will be compared across all 3 groups.

Due to some uncertainty about the power of the study as described above, we have the following backup plan. After enrolling 30 patients, we plan to submit the primary outcome data, together with the sealed code for unblinding the data, to a statistical consultant who will not otherwise participate in the study. If statistically significant differences in efficacy are apparent among the 3 arms at this point (*i.e.*, $p < 0.01$, ANOVA), we will conclude enrollment and further analyze the results. Otherwise we will seek to enroll an additional 5 subjects in each arm before we are informed of the study code.

E. Patient Assignment to Treatment

The order of assignment to one of the three treatment arms will be random.

F. Dosage and Administration

See the summary table in III.A. for an overview. Patients who meet entry criteria will take identical study tablets nightly, containing either 5mg or 10mg olanzapine or placebo. Due to concerns about the tolerability of starting olanzapine abruptly at the 10mg qhs dose, we plan to administer only 5mg nightly for the first week of the "10mg" arm.

1. Materials and Supplies

We will use matching dose cards, one for each week of the study, each with 9 days' supply of drug. The last 2 days will be clearly marked as extras, and will be used to better assess compliance and in the event that a patient is a day or two late returning to clinic. Each card will be marked "Week 1", "Week 2", etc., as appropriate. Each card will also be marked with a code (e.g. A, B, or C) which will indicate which study arm is involved. Here, "A", "B", and "C" refer to the placebo, 5mg, and 10mg arms, but of course not necessarily in that order. We will also ask Lilly to supply us simultaneously with a sealed envelope containing the code, *e.g.* A=placebo, etc. This envelope will remain sealed until 30 patients have been enrolled, as described above.

G. Blinding

The study will be conducted in a double-blind fashion. See III.F.1., immediately above.

H. Concomitant Therapy

Concomitant therapy of Parkinson's disease including levodopa, carbidopa, selegiline, and dopamine agonists will be allowed if it satisfies the Inclusion and Exclusion Criteria given in section III.D.1. above. However, we will make every reasonable effort not to *change* the dose of any CNS-active medication during the 4 weeks of this study. The exception will be when we allow for upward titration of dopamimetics at the 2-week visit, if indicated.

I. Efficacy and Safety Evaluations

Please see the information following the table in III.A. above for the timing of outcome measures. These fall into the following categories:

Primary and Secondary Efficacy Measures

Primary: blinded BPRS ratings of psychosis; Clinical Global Impression/Improvement scores
Secondary: PDQ-39 quality of life scores, ADL assessments (Schwab-England, UPDRS), sleep log, and Beck depression rating scales

1,2. Safety and Safety Monitoring

The primary safety measures will be UPDRS motor ratings, sleep log, and MMSE, in addition to a clinical Review of Systems on each visit. The Review of Systems is integrated into our routine patient care database. Significant **adverse events** will be reported to Lilly on standard forms. No routine clinical laboratory tests are anticipated.

3. Drug Accountability

A pill count will be performed on each Week 2 or Week 4 visit. Records will be kept for each patient of total pills used.

IV. Data Analysis Methods (including any interim analyses)

The 0-2 week and 2-4 week periods will be treated as two separate study periods for the purpose of statistical analysis, due to the possible addition of dopamimetics at the 2-week visit; the change from 0 to 2 weeks will be the primary test of efficacy. If we have few dropouts, a repeated-measures ANOVA with post-hoc tests would be adequate and powerful (dependent variable: BPRS positive symptom subscale scores; grouping variable: dose; within variable: time). Otherwise a more complicated actuarial analysis might be needed. We have collaborated in the past with the biostatistics group here at Washington University. Investigators will remain blinded throughout accrual and treatment. See section III.D.2. above for a preliminary analysis of data, after treatment of 30 subjects, by a statistical consultant not otherwise participating in the study.

V. Informed Consent, Ethical Review, and Regulatory Considerations

See attached documents submitted to the Washington University School of Medicine Human Studies Committee.

Since olanzapine has an FDA indication for treatment of "psychotic disorders," the use of olanzapine to treat psychosis in this trial is considered to be within the scope of the current labeling. Also, given the positive tolerability data from the open study in PD, (Wolters et al., 1996), we do not feel that use of olanzapine in this population constitutes any additional risk.

However, since it is possible that data from this study may eventually be used to support a request to the FDA for a change in labeling or advertising for olanzapine, we are applying to the FDA for an Independent IND approval.

Reference

Wolters, E. C. et al. (1996). "Olanzapine in the treatment of dopaminomimetic psychosis in patients with Parkinson's disease." Neurology **47**: 1085-1087.
