# Supplementary material for: A fixed-dose randomized controlled trial of olanzapine for psychosis in Parkinson disease
Source: F1000Res. 2013 Jul 9;2:150. [Version 1] doi: 10.12688/f1000research.2-150.v1 (PMC3907164; doi:10.12688/f1000research.2-150.v1)
Supplement: Peer review history

# Peer review: Referee response for version 1

**Published:** 2013-08-22

**Authors:** Eric Molho

**Affiliations:** 1 Department of Neurology, Albany Medical Center, NY, USA

This is a well conceived and rigorously carried out clinical trial addressing an important issue in a difficult to study patient population. Although the data was collected a decade ago, the information is still relevant because remarkably little progress has been made since 2003 in the treatment of PD related psychosis. Dr. Richard’s referee response presents the methodological shortcomings and correctly points out resulting limitations in data interpretation. I agree with all of the comments in her review.

In addition, I would like to add the following comments/observations: 

In the abstract and introduction, the authors suggest that because olanzapine has a similar receptor binding profile to clozapine, it is a good candidate drug to study in the PD population and they cite Seeman *et al. *1993. More recent work by Seeman shows that what is unique about clozapine is that it binds D2 receptors “loosely” and that its receptor occupancy is short-lived. Quetiapine has a similar D2 binding profile to clozapine but, olanzapine behaves much more like haloperidol and risperidone in this regard. This may explain the motor worsening that seems to be associated with olanzapine use in PD. ( Kapur and Seeman. Am J Psychiatry 2001;158:360-369. Seeman and Tallerico. Am J Psychiatry 1999;156:876-884.)

I disagree with the statement in the introduction that “ *Delusions, when they occur, often antedate visual hallucinations...*.” The natural history of hallucinations in PD has been looked at in only a few longitudinal studies and the interaction between hallucination and delusions has not been adequately studied but my clinical experience and the bulk of what is presented in the literature suggests that minor hallucinatory phenomena (illusions, passage or presence hallucinations, benign hallucinations with insight) are much more common and occur earlier in PD than delusions. Delusions are generally associated with more severe underlying cognitive impairment, by definition are associated with lost insight and are generally considered to indicate a more advanced stage of disease progression than isolated visual hallucinations. ( Fenelon *et al. *Brain 2000;123:733-745).

I would also question the premise that allowing for a dopaminergic medication dose adjustment in the study protocol (to reverse any worsening of PD motor symptoms due to the introduction of low dose olanzapine) might produce study results that are a more appropriate way to evaluate how this drug might work in the clinic. My experience is that this strategy is unsustainable even in the short term in this population. One reason is that each dose increase of dopaminergic medication is prone to reignite symptoms of psychosis and thus lead to a need for higher doses of the antipsychotic, setting up a cycle of overall worsening. Secondly, these patients tend to have more advanced motor symptoms including high risk symptoms such as postural instability and dysphagia. It may be very difficult to continue a drug known to worsen PD motor symptoms after the first fall or choking episode even if one cannot be sure it was a direct result of the antipsychotic drug.   

I would suggest more clearly pointing out that this study cohort had relatively mild cognitive impairment (MMSE >26) which may be quite different than what is encountered in practice. More demented patients may be more susceptible to side effects of atypical neuroleptics including sedation, encephalopathy and motor worsening. More demented patients may also have more severe psychosis which is likely to be less responsive to the low doses used in this and some other clinical trials.  

Finally, I suspect that some readers may not have a full grasp of “Type II error”. This should probably be spelled out without jargon in the discussion.

I have read this submission. I believe that I have an appropriate level of expertise to confirm that it is of an acceptable scientific standard.

# Response

**Published:** 2013-08-22

**Authors:** Kevin J. Black

**Affiliations:** Washington University in St. Louis, USA

Dr. Molho's comments are well thought out and much appreciated. 

I agree with the discussion about clozapine pharmacology with reference to higher dissociation rate/decreased binding avidity as a better explanation for clozapine's superior tolerability in PD.

I agree that delusions usually follow hallucinations in this population; my error.  

Dr. Molho's comments on mid-protocol antiparkinsonian dose adjustment are reasonable. Nevertheless the rationale described explains the original protocol design, and as he points out, we need more controlled data to definitively answer this and other questions about treatment.  

I agree that the mild cognitive impairment in this sample limits the application of the results to a total clinical population of psychosis in PD. However, it can also be seen as a strength of the study, in that psychosis with dementia may conceivably differ in etiology and optimal treatment, so in that respect this sample may be considered more "pure."

# Peer review: Referee response for version 1

**Published:** 2013-07-11

**Authors:** Irene Richard

**Affiliations:** 1 University of Rochester, Rochester, NY, USA

The study (completed in 2003) was well designed, albeit with a relatively small sample size, and intended to answer an important and clinically relevant question.  After reviewing the manuscript I would agree that it probably does support the notion that olanazapine may cause intolerable worsening of motor disability but I do not think that one can draw any conclusions regarding efficacy or lack thereof based on this study.  

 

One element of the trial design (i.e. permitting changes in dopaminergic medications) may have created some challenges when interpreting the data.  The investigators speculated that olanzapine may be better tolerated if adjustments in dopaminergic medications were allowed (and therefore permitted dopaminergic medication adjustment at the 2 week visit).  While this may be true, it could also increase the chances that dopaminergic drug induced psychosis could worsen (if dopaminergic medication were increased in an effort to improve motor worsening).  This could potentially be a "set up" for decreased efficacy (if dopaminergic medications were changed more frequently in active vs. placebo).  It is noted that medications were adjusted in one of the placebos, two of the 2.5 mg active and one of the 5 mg active.  The authors note that there was an apriori decision to analyze data from weeks 0-2 and 2-4 separately.  Change from 0-2 weeks was chosen to be the primary test of efficacy, apparently in order to limit the confound of changes in dopaminergic medications allowed at week two.  However, one could question if 2 weeks is long enough to demonstrate efficacy.   

 

In addition, a series of unplanned events contributed to challenges with data interpretation. These events included a change in design after study initiation, lower than expected enrollment and high dropout rate.

 

The change in study design was a decrease in study drug dosage after enrollment of 5 subjects ("in light of reports published since study initiation that higher olanzapine doses cause intolerable exacerbation of parkinsonism in PD").  This resulted in one subject being excluded from analyses (see below) and perhaps, decreased the chance of demonstrating study drug efficacy (if higher dosages were required).  

 

Only 24 (of an anticipated 30) subjects were enrolled and 9 withdrew (39%) which is a fairly high dropout rate.  One of the 24 subjects was not included in the analyses because he was the only one to receive the initially planned dosage of 10 mg.  

 

While spontaneous reports of motor side effects were not statistically significant between groups, a disproportionate number of olanzapine vs. placebo who withdrew did so due to motor side effects.  This finding does suggest that olanzapine may be associated with worsening motor function.  However this may not be true for every patient, as exemplified by the one subject who was the only to receive the initially planned dosage of 10 mg.  He had no worsening of motor function (and an improvement in psychosis).

I have read this submission. I believe that I have an appropriate level of expertise to confirm that it is of an acceptable scientific standard.

# Response

**Published:** 2013-07-11

**Authors:** Kevin J. Black

**Affiliations:** Washington University in St. Louis, USA

I agree with Dr. Richard's comments about the limitations and possible conclusions from this report.
